# Laser Energy Application in Endoscopic Kidney-Sparing Surgery for Upper Tract Urothelial Carcinoma: A Systematic Review of Oncological Outcomes and Surgical Complications

**DOI:** 10.3390/cancers18050821

**Published:** 2026-03-03

**Authors:** Federico Zorzi, Pietro Scilipoti, Stefano Moretto, Carlos Gonzalez-Gonzalez, Nicola Nannola, Daniele Robesti, Andrea Folcia, Marie Chicaud, Stessy Kutchukian, Luigi Candela, Berthe Laurent, Eugenio Ventimiglia, Francesco Montorsi, Alberto Briganti, Andrea Salonia, Luca Villa, Steeve Doizi, Olivier Traxer, Frédéric Panthier

**Affiliations:** 1 Service d’Urologie, Tenon Hôpital, Rue de la Chine 4, 75020 Paris, France; 2 Endolase Laboratory, GRC20-Sorbonne University, PIMM-Arts et Métiers Paris Tech, 75013 Paris, France; 3 Urology Unit, Department of Medical, Surgical and Health Sciences, University of Trieste, 34127 Trieste, Italy; 4 Unit of Urology, Department of Experimental Oncology, URI, IRCCS Ospedale San Raffaele, 20132 Milan, Italy; 5 Ospedale San Raffaele, Vita-Salute San Raffaele University, 20132 Milan, Italy; 6 Department of Urology, Humanitas Clinical and Research Institute IRCCS, 20089 Rozzano, Italy; 7 Department of Urology, University of Modena and Reggio Emilia, 41121 Modena, Italy; 8 Department of Urology and Kidney Transplantation, Centre Hospitalier Universitaire, 86021 Poitiers, France; 9 Department of Surgical Sciences, Uppsala University, 75310 Uppsala, Sweden

**Keywords:** upper tract urothelial carcinoma, kidney-sparing surgery, endoscopic laser ablation, oncological outcomes, complications

## Abstract

**Background:** Endoscopic kidney-sparing surgery (eKSS) is increasingly adopted for the management of selected patients with upper tract urothelial carcinoma (UTUC). Laser energy is central to tumor ablation during eKSS; however, multiple laser platforms with distinct physical and thermal properties are currently available, and their comparative oncological and safety profiles remain poorly defined. This systematic review aims to summarize the available evidence on oncological outcomes and perioperative complications associated with laser-based endoscopic treatment of UTUC and to explore potential differences according to laser technology. **Methods:** A systematic literature search identified 25 eligible studies published between 1997 and 2024, including 1344 patients treated with laser-assisted eKSS. All included studies were non-randomized, predominantly retrospective, and characterized by moderate-to-serious risk of bias. Holmium:YAG, Thulium:YAG (thu:YAG, continuous-wave and pulsed), thulium fiber laser (TFL), Neodimio:YAG (Nd:YAG), diode lasers, and combination platforms were reported. **Results:** Ipsilateral upper tract recurrence was common across all laser categories, with weighted proportions ranging approximately from 27% to 52% and substantial inter-study heterogeneity. Progression and conversion to radical nephroureterectomy (RNU) were relatively infrequent overall, with numerically weighted proportions observed in thu:YAG-based cohorts. Major complications (Clavien–Dindo ≥ III) were rare across all laser technologies, although a trend toward a higher weighted proportions was observed in Ho:YAG- and Nd:YAG-based series. Minor complications were more frequently reported and highly heterogeneous. **Conclusions:** Available evidence supporting laser selection in endoscopic kidney-sparing management of UTUC is limited and largely descriptive. Thulium:YAG and TFL platforms seem to demonstrate encouraging trends toward lower progression and conversion to-radical-nephroureterectomy rates; however, these findings are derived from heterogeneous, non-comparative studies with limited follow-up. No standard laser platform can currently be recommended over others based on existing data. Prospective, comparative, and methodologically robust studies are required to determine whether laser technologies confer clinically meaningful advantages in oncological control or safety for UTUC treated with eKSS.

## Simple Summary

Endoscopic kidney-sparing surgery (eKSS) has become an established treatment option for selected patients with upper tract urothelial carcinoma (UTUC), aiming to preserve renal function while maintaining acceptable oncological control. Laser energy represents the cornerstone of endoscopic tumor ablation, yet multiple laser platforms with different physical properties are currently available, and their relative impact on oncological and safety outcomes remains unclear. This systematic review summarizes the available clinical evidence on laser-based endoscopic management of UTUC, focusing on recurrence, progression, conversion to radical nephroureterectomy, and treatment-related complications. Overall, recurrence after eKSS remains frequent across all laser technologies, whereas progression and major complications are uncommon. Thulium-based lasers and thulium fiber laser (TFL) appear to have lower progression and radical surgery rates compared with holmium:YAG in descriptive analyses, although follow-up is generally shorter and evidence is limited by study heterogeneity and retrospective designs. Current data do not support a definitive superiority of one laser technology over another. Treatment selection should therefore be guided by tumor characteristics, surgeon expertise, and institutional experience. Well-designed prospective and comparative studies are urgently needed to clarify whether specific laser platforms translate into meaningful oncological or safety advantages in kidney-sparing UTUC management.

## 1. Introduction

Upper tract urothelial carcinoma (UTUC) represents approximately 5–10% of urothelial malignancies and remains a therapeutic challenge due to pronounced biological heterogeneity and the functional consequences of radical treatment [1,2].

Radical nephroureterectomy (RNU) is the reference standard for high-risk disease [3]; however, according to EAU guidelines, endoscopic kidney-sparing surgery (eKSS) has become the treatment of choice in patients presenting low-risk tumors and in imperative settings such as solitary kidney, bilateral disease, or end-stage renal disease [4,5].

Progresses in ureteroscopic technology, growing surgical expertise, enhanced knowledge of oncological outcomes, and standardized follow-up strategies have collectively transformed conservative management from a historical alternative into an established pathway for selected patients in specialized centers [6].

Nonetheless, endoscopic eKSS is characterized by substantial rates of local recurrence and repeated procedures, and outcomes appear strongly influenced by operative performance [7]. Historically, different energy sources were employed for the treatment of upper tract tumors, while the advent of laser energies has consented an evolution in terms of precision surgical control, implementing ablation efficiency and safety [8]. Laser platforms differ in wavelength, emission mode (pulsed vs. continuous), tissue absorption, coagulation capacity, and thermal penetration depth, potentially affecting bleeding control, visualization, urothelial injury, and completeness of ablation [9]. Holmium: Yttrium-Aluminum-Garnet (Ho:YAG) has traditionally been widely used due to its shallow penetration and versatility, whereas thulium-based platforms, traditionally referred to continuous-wave thulium (Tm-YAG), pulsed thulium fiber systems (TFLs), and more recently Thulium: Yttrium-Aluminum-Garnet (p-Tm:YAG), offer enhanced photothermal coagulation, potentially improving precision and minimizing bleeding [10].

Despite a rapidly evolving device landscape, clinical evidence comparing different laser energies in UTUC remains scarcely fragmented [11]. Currently available data do not provide clear guidance regarding which laser platform should be preferred, particularly in light of the wide range of available technologies and the heterogeneity of tumor risk profiles [12].

Accordingly, this systematic review aims to evaluate oncological and safety outcomes of endoscopic conservative management of UTUC performed using laser energy sources and to explore whether specific laser modalities are associated with differential outcomes. Through this focused synthesis, we sought to summarize the available evidence, explore heterogeneity according to laser technology, identify knowledge gaps requiring prospective validation, and highlight descriptive trends that may inform surgical practice in the kidney-sparing endourological setting.

## 2. Methods

### 2.1. Protocol Registration and Reporting Standards

This systematic review was conducted according to the predefined protocol registered in the PROSPERO database (N. CRD420251269475). Data were reported in accordance with the Preferred Reporting Item for Systematic Review and Meta-Analysis (PRISMA) statement [13].

### 2.2. Research Strategy and Study Selection

A comprehensive literature search was performed in three electronic databases (MEDLINE/PubMed, Embase, and Scopus) from 1 January 2000 to 31 December 2025. The detailed search strategies for each database are provided in the Supplementary Files S1 and S2. Search terms combined controlled vocabulary and free-text keywords related to upper tract urothelial carcinoma, ureteroscopy, endoscopic conservative management, and laser technologies (e.g., Ho:YAG, TFL, Tm:YAG, p-Tm:YAG, and Nd:YAG). Reference lists of eligible articles and relevant reviews were manually screened to identify additional studies.

After duplicate removal, title and abstracts were screened independently by two reviewers. Full-text articles of potentially eligible studies were subsequently assessed according to predefined inclusion and exclusion criteria. Disagreements were resolved by consensus a third voter (F.P.).

### 2.3. Eligibility Criteria

Randomized clinical trials, prospective or retrospective comparative cohort studies, and single-arm cohort studies reporting outcomes of endoscopic conservative management of UTUC performed using laser energy were eligible. Case reports, small case series including fewer than ten patients, narrative reviews, editorials, letters, conference abstracts without full text, and experimental or animal studies were excluded.

Eligible studies enrolled adult patients with histologically confirmed UTUC treated with kidney-sparing endoscopic approaches.

Only studies in which the laser energy source or technology was clearly specified were included. Laser technologies of interest comprised Ho:YAG, Thulium:YAG systems, thulium fiber laser (TFL), and other explicitly reported laser energies such as Nedimio: Yttrium-Aluminum-Garnet (Nd:YAG) or diode lasers. Studies were excluded if the laser type was not reported, if conservative treatment involved mixed non-laser modalities (e.g., Bugbee or electrocautery) without separable laser-only outcome reporting, or if outcome data were not extractable for the laser-treated subgroup.

Comparators were not mandatory. When present, comparators included alternative laser energies or alternative kidney-sparing strategies. Studies including RNU comparator arms were eligible provided that laser-specific eKSS data could be extracted.

### 2.4. Outcomes

The primary outcome was ipsilateral upper tract recurrence following endoscopic laser treatment. Secondary outcomes included progression (as defined by individual authors), conversion to RNU, intravesical recurrence, recurrence-free survival, disease-specific survival, overall survival, and safety outcomes, including overall complications, major complications (Clavien–Dindo grade ≥ III), ureteral strictures, bleeding requiring transfusion or intervention, infection or sepsis, and hospital readmission.

Progression was defined according to authors’ definitions and included one or more of the following: histological upgrade (low-grade to high-grade), development of muscle-invasive disease, development of metastatic disease, tumor becoming no longer amenable to conservative endoscopic management, or conversion to radical treatment due to biological progression.

### 2.5. Data Extraction

Data were extracted using a predefined standardized extraction template. Extracted variables included study characteristics (design, setting, and study period), patient demographics, tumor characteristics (grade, risk profile when available, size, multifocality, and location), endoscopic technique and instrumentation (flexible versus semirigid ureteroscopy, ureteral access sheath use, and percutaneous access), laser characteristics (technology, fiber diameter, and power/settings when available), oncological outcomes, survival endpoints, and safety outcomes.

When outcomes were reported as Kaplan–Meier estimates, 2- and/or 5-year survival rates were extracted. In multi-arm studies (e.g., Ho:YAG versus Tm:YAG), each laser arm was extracted separately. When data were incomplete or unclear, outcomes were recorded as not reported.

### 2.6. Risk of Bias Assessment

Risk of bias was assessed using the ROBINS-I (version developed in 2016) tool for non-randomized studies. If randomized trials were identified, the RoB 2 tool (version developed in 2019)was planned for use. Studies were categorized as having low, moderate, serious, or critical risk of bias.

### 2.7. Laser Technology Classification

Laser technologies were classified into predefined categories based on the reported energy source: Ho:YAG alone; Thu:YAG alone (including continuous-wave Tm:YAG and p-Tm:YAG systems, excluding TFL); Nd:YAG alone; TFL alone; and combination platforms (e.g., Ho + Thu, Nd/Ho). Insufficiently specified technologies were labeled as other/unspecified.

### 2.8. Statistical Analysis and Data Synthesis

Given the substantial clinical and methodological heterogeneity across included studies (differences in study design, patient selection and risk profile, tumor characteristics, endoscopic techniques, definitions of outcomes, and follow-up schedules), we performed descriptive synthesis and did not conduct a formal meta-analysis.

Laser technologies were extracted from each study and classified into pre-specified categories using a rule-based algorithm applied to the reported laser type. Specifically, studies were grouped as Ho:YAG alone, Thu:YAG alone (including both continuous-wave and p-Tm:YAG systems, excluding thulium fiber laser), Nd:YAG alone, TFL alone, and combination categories (e.g., Ho + Thu, Ho + TFL, and Nd/Ho), with remaining or insufficiently specified technologies labeled as Other/Unspecified.

For each outcome, we reported study-level proportions calculated as the ratio between the number of events and the corresponding denominator reported in the original study (events/N). Outcomes included oncological endpoints (recurrence, progression, and RNU) and safety endpoints (any complications and major complications defined as Clavien–Dindo ≥ 3).

For descriptive summary estimates within each laser category, we calculated a weighted overall proportion as the ratio of aggregated counts across studies:

(1) $${\hat{p}}_{\mathrm{weighted}}=\frac{\sum_{i}{\mathrm{events}}_{i}}{\sum_{i}N_{i}}$$

This approach provides a transparent, sample-size-weighted descriptive estimate without modeling between-study variability. Exact 95% confidence intervals for both study-level and weighted overall proportions were computed using the Clopper–Pearson method based on binomial counts.

For oncological outcomes, we additionally summarized follow-up time. Study-level follow-up (months) was extracted as reported; entries reported as “not reported” were treated as missing. Within each laser category, the follow-up corresponding to the descriptive overall estimate was summarized using the weighted median follow-up, weighting each study by the same denominator used for the outcome (i.e., the study’s N contributing to that endpoint). Descriptive results were visualized using forest-style plots displaying study-level proportions for each laser category, an “Overall (weighted)” row reporting the aggregated proportion and its confidence interval; follow-up was displayed for oncological endpoints.

Statistical significance was considered at *p* < 0.05. For all statistical analyses, R software environment for statistical computing and graphics was used (version 4.5.1).

## 3. Evidence Synthesis

### 3.1. Study Selection and Overall Characteristics

**Figure 1:**
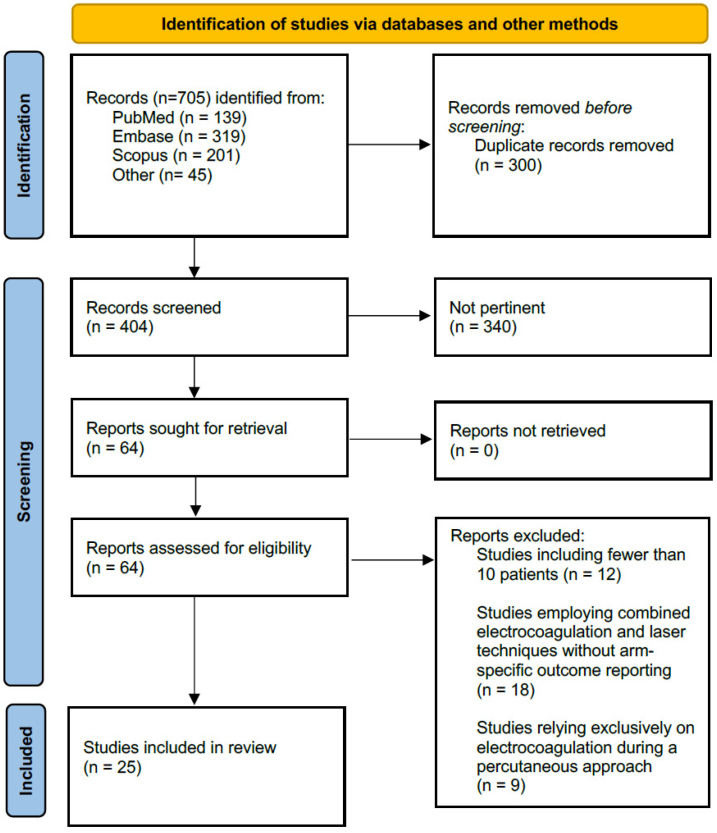
PRISMA flow diagram illustrating the study selection process for inclusion in the systematic review.

After screening 705 records, 25 studies met inclusion criteria (Figure 1). Most included studies were single-center retrospective cohorts or case series. Three studies were multicenter retrospective cohorts [14,15,16], and five studies had a prospective design [17,18,19,20,21]. No randomized controlled trials were identified. One prospective study included a matched control group [18].

Patients were treated over a period spanning from 1997 to 2024. Sample size ranged from 13 to 139 patients per study. Overall, the 25 included studies comprised 1344 patients undergoing kidney-sparing endoscopic management of UTUC using laser-based techniques.

### 3.2. Risk of Bias

**Figure 2:**
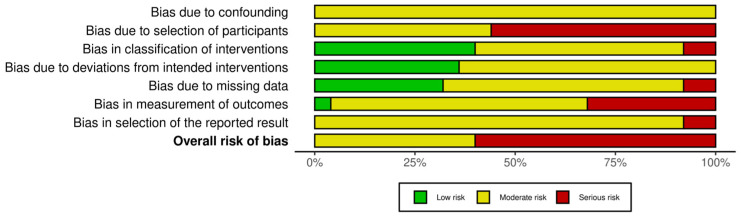
Risk of bias by domains across all studies.

Risk of bias assessment revealed overall moderate-to-serious methodological limitations across the included studies (Figure 2 and Supplementary Figure S1). The predominant sources of bias were related to patient selection and study design, as most studies were retrospective, single-center cohorts or case series without randomization or control groups.

Bias related to classification of interventions was low, as laser technology and treatment modality were clearly specified in most reports. Bias arising from deviations from intended interventions and missing outcome data was judged to be low to moderate in the majority of studies. Heterogeneity derived from measurement of oncological outcomes, which were typically based on standard endoscopic, radiological, and histopathological assessments. Furthermore, inconsistent definitions, follow-up schedules, and reporting thresholds introduced additional uncertainty.

No study was judged to be at low risk of bias across all domains. The overall certainty of evidence should therefore be considered limited, supporting the choice of descriptive, non-meta-analytic synthesis.

### 3.3. Patient Characteristics

Overall, 345 patients were treated using Ho:YAG laser energy, while 418 patients received thulium-derived technologies, including 239 treated with Tm:YAG systems and 179 with TFL.

Mixed Ho:YAG—Tm:YAG platforms were used in 246 patients. Nd:YAG alone was employed in 65 patients, Nd:YAG combined with Ho:YAG in 122 patients, and diode laser systems in 15 patients.

Across included studies, mean patient age was 70 years, with reported mean or median ages ranging from 55.8 to 77.4 years. The overall proportion of male patients was approximately 71%. Ho:YAG-based series reported mean ages of 69–70 years, Thu:YAG-based series 71 years, and TFL-based series 72–73 years, while combination-laser cohorts demonstrated similar age distributions.

#### 3.3.1. Pathology Features

Three studies included exclusively patients with tumors located in the renal collecting system [16,21,22], whereas the remaining studies enrolled patients with mixed renal and ureteral involvement. Multifocal disease was reported in approximately 12% to 45% of patients per series.

Among studies providing numerical distributions, approximately half of tumors were located in the renal pelvis or calyceal system, 35–45% in the ureter, and 10–20% showed combined intrarenal and ureteral involvement [15,16,23,24,25,26,27,28].

High-grade disease and/or high-risk UTUC constituted a substantial proportion of treated cases. Among studies reporting histological grade, the proportion of high-grade tumors ranged from 8% to 74% [15,16,19,20,21,23,24,25,26]. Among studies providing UTUC risk classification, high-risk tumors accounted for 40% to 53.4% of included lesions, and in one study all patients were classified as high risk [15,20,25,27,28,29] (Table 1).

#### 3.3.2. Endoscopic Technique

Ureteroscopy was the primary treatment modality in the majority of cohorts. Flexible ureteroscopy was the main approach in 18 studies [14,15,16,20,21,22,23,24,25,26,27,28,29,30,31,32,33,34]. No series reported exclusive use of semirigid ureteroscopy, which was used only in combination with flexible instruments. One study reported exclusive use of a percutaneous approach [17], while six studies reported ureteroscopy without specification of instrument type [21,29,31,34,35,36].

**Table 1:** Baseline characteristics of 25 studies included in the systematic review.

Study	Country	Design	Study Period	Number of Patients	Age	Male (%)	Follow-Up (Months)	Tumor Location	Multifocality (%)	High Grade/High Risk (%)	Approach Type (%)
**Defidio (2019)** **[18]**	Italy	Single center; retrospective cohort	2005–2018	101	71.05 ± 10.32	79.2%	28.7 (29.4)	Mixed (above UPJ 42.6%, below UPJ 39.6%, both 17.8%)	multiple 36.6%	High-grade 18.8% (ITT)	mixed URS (semirigid + fURS)
**Niţă (2012)** **[37]**	Romania	Single center; retrospective cohort	1998–2011	65	67 (42–89)	NR	60 (6–120)	Renal pelvis/calices 39 (60%), ureter 26 (40%)	multiple 27.7% (18/65)	High-grade 32.3% (21/65)	mixed URS and percutaneous
**Defidio (2011)** **[38]**	Italy	Single center; case series (observational)	2005–2009	59	66	66.1%	26.4	mixed: intrarenal 30/59 (50.9%), ureteral 13/59 (22%), combined 16/59 (27.1%)	multiple 55.9% (33/59)	NR	mixed URS (semirigid + fURS)
**Tada (2010)** **[14]**	Japan	Multicenter; case series	2004–2007	15	75 (51–86)	73.3%	25.5 (13–51)	Predominantly ureter (most U2/U3, 1 renal pelvis case)	NR	High grade (G3) 2/15 = 13.3%	fURS
**Li (2025)** **[17]**	China (Beijing Chaoyang Hospital, Capital Medical University)	Single center; prospective cohort study	2017–2020	30 (15 PC laser ablation; 15 RNU)	65.5 (6.1)	70%	34 (10–54)	Renal pelvis only (inclusion criteria)	0	High grade: PC 6/15 (40%); RNU 7/15 (47%)	percutaneous
**Geavlete (2024)** **[22]**	Romania	Single center; prospective comparative cohort (matched control group)	2015–2018	122	NR	NR	36 (3-y outcomes)	Pyelocaliceal UTUC (pTa only)	NR	High grade pTa: 5/61 (8.2%) in each arm	fURS
**Hsieh (2020)** **[25]**	Taiwan	Single center; Retrospective cohort	2012–2018	34	71 (39–89)	29.4%	NR	Ureter 62%, renal pelvis 38%	NR	High grade 74% (25/34)	mixed URS (semirigid + fURS)
**Wen (2018)** **[28]**	China	Single center; retrospective comparative cohort	2013–2017	139 (32 tm:YAG +107 RNU)	tm:YAG 69.3 (11), RNU 62.3 (9.4)	tm:YAG 65.6%, RNU 71%	NR	Predominantly ureter (tm:YAG 28/32; RNU 78/107)	NR	tm:YAG high-grade 5/32 (15.6%); RNU high-grade 32/107 (29.9%)	mixed URS (semirigid + fURS)
**Territo (2025)** **[27]**	Spain (Barcelona)	Single center; retrospective cohort (single-arm)	2022–2024	33	74 (IQR 70–83)	75.8%	NR (minimum FU 6mo)	Renal pelvis only (inclusion criteria)	21% multiple locations (7/33), 30% multiple lesions on CT (10/33)	High-grade 71% (20/28 UC biopsies)	mixed URS (semirigid + fURS)
**Villa (2018)** **[19]**	France	Single center; prospective database, retrospective analysis (single-arm cohort)	2003–2016	92	69.7 (34.5–90.8)	67.4% (62/92)	52.4	Renal collecting system 62% (57/92)	46.7% (43/92) multifocal	High-grade 14.1% (13/92), grade NR in 47.8%	fURS
**Proietti (2022)** **[26]**	Italy	Single center; retrospective case series	2021–2022	28	73 (6.2)	75% (21/28)	NR (endoscopic FU 2/6/12)	Mixed	42.9% (12/28 multifocal)	High risk group 53.6% (15/28); high-grade biopsy 28.6% (8/28)	Mixed URS (semirigid + fURS)
**Bozzini (2021)** **[15]**	Italy	Multicenter; retrospective cohort	2015–2016	78	69.2 (12.8)	NR	11.7 (9.9)	Renal 89.7% (70/78)	16.7% (13/78 multiple tumors)	High grade 37.2% (29/78)	fURS
**Matsuoka (2003)** **[32]**	Japan	Single center; retrospective case series	NR	30 patients (33 procedures)	NR	NR	37mo (imperative), 33mo (elective)	Ureteral lesions 20/33 procedures (60.6%)	Multiple tumors: imperative 4/7 vs. elective 1/20	High grade poorly reported; biopsy grade known only in subset	Mixed URS (semirigid + fURS)
**Ye (2025)** **[20]**	China	Single center; prospective pilot single-arm study	2021–2024	33	75 (66–82)	58% (19/33)	23 (18–39) for pz without death/salvage RNU	Renal pelvis 45% (15/33)	48% (16/33)	All high-risk UTUC; high grade 55% (18/33); hydronephrosis 73%	Mixed URS (semirigid + fURS)
**Cornu (2010)** **[16]**	France	Multicenter; retrospective cohort	2003–2007	35	67 (13.1)	94.2%	30 (12–66)	Kidney cavities 54.3% (19/35)	34.3% (12/35 multiple)	High grade 17.1%, stage pT1 2.9%, grade/stage unavailable 37%	fURS
**Hubosky (2013)** **[31]**	USA	Single center; retrospective cohort	1995–2010	13	56.5 (38–73)	53.8%	59 (10–188)	Ureter 67% (10/15)	NR	High grade (1973 WHO): 2/15 (13%); (2004 WHO applied retrospectively): high grade 4/15 (27%)	URS (not specified)
**Kalaitzis (2013)** **[21]**	Greece	Single center; prospective single-arm series	2000–2011	25	71 (9)	52%	6 (3–68)	NR	0.44	8% high grade (2/25 G3)	URS (not specified)
**Musi (2018)** **[23]**	Italy	Single center; retrospective cohort	2012–2016	42	68 (10.7)	71.4% (30/42)	26.3 (2–54)	Renal pelvis 31%; lower ureter 35%	12% (5/42)	High grade 9.5% (4/42); CIS 2.4% (1/42); “high-risk” included only in imperative cases	fURS
**Proietti (2021)** **[34]**	Italy	Single center; retrospective cohort	2013–2019	29	69 (63–79)	75.9% (22/29)	23 (14–35)	Kidney 65.5% (19/29)	34.5% (10/29 multiple)	High grade 62.1% (18/29)	URS (not specified)
**Sanguedolce (2021)** **[30]**	Spain	Single center; retrospective cohort	2015–2019	47	75 (67–81)	74.5% (35/47)	24 (17–44)	Pelvis/calyces 53.2% (25/47)	31.9% (15/47)	High grade 17.0% (8/47); high-risk 61.7% (29/47)	fURS
**Shvero (2021)** **[33]**	Israel	Single center; retrospective cohort	2014–2019	62	70 (65–75)	69.5% (41/59)	22/11–41)	Renal pelvis/calyces 54.8% (34/62 renal units)	27.4% (17/62 renal units)	Low-grade only (inclusion criterion)	fURS
**Yoshida (2021)** **[24]**	Japan	Single center; retrospective comparative cohort	2006–2019	26:10 PDD Th:YAG + Ho:YAG), 16 Ho:YAG	77.7 (71.3–83.3)	63.1%	NR	Renal pelvis 37.5%; ureter 62.5%	20.6% multifocal	High grade on biopsy: PDD 40% (4/10); Ho 56.2% (9/16); imperative/relative indications 80% vs. 87.5%	fURS
**Figaroa (2024)** **[35]**	Netherlands	Single center; retrospective cohort	2010–2020	71	67.5 (57/71)	80% (21.5–68.3)	49.5 (IQR 21.5–68.3)	Renal pelvis 36% (26/71), distal ureter 35% (25/71)	Multifocal 4.2% (3/71)	High-grade at diagnosis 19% (13/71); low-grade 81% (58/71)	URS (not specified)
**Proietti (2025)** **[29]**	Italy	Single center; retrospective case series	2024	20	74.5 ± 8.5	65% (13/20)	Short-term; 2–12 mo protocol	NR	NR	High-risk 40% (8/20); HG 40% (8/20)	URS (not specified)
**Bislev (2024)** **[36]**	Denmark	Single center; retrospective cohort	2012–2022	118	75 (69–81)	69.5%	36 (20.3–58.8)	NR	NR	High-risk 0/118 (0%)	URS (not specified)

Abbreviations: UTUC, upper tract urothelial carcinoma; URS, ureteroscopy; fURS, flexible ureteroscopy; UPJ, ureteropelvic junction; RNU, radical nephroureterectomy; ITT, intention-to-treat; CIS, carcinoma in situ; FU, follow-up; IQR, interquartile range; NR, not reported; PC, percutaneous; ETLT, endoscopic thulium laser treatment; HG, high grade; LG, low grade; PDD, photodynamic diagnosis; CT, computed tomography; UC, urothelial carcinoma.

#### 3.3.3. Laser Technologies, Settings and Technical Parameters

**Figure 3:**
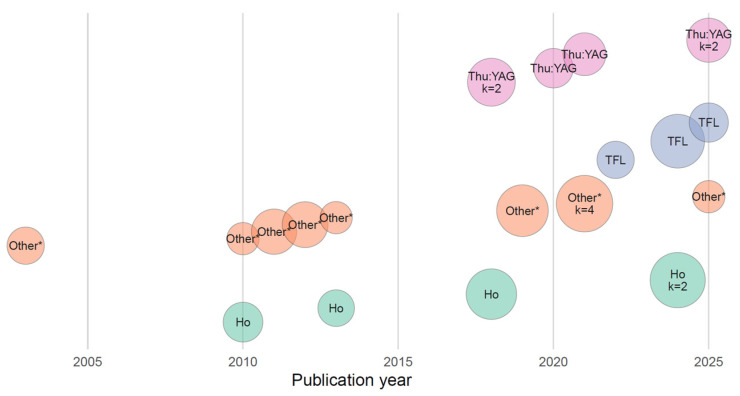
Temporal distribution of laser technologies reported in the studies included in the systematic review. The Other* category includes laser systems not classifiable as Ho:YAG, Thu:YAG, or thulium fiber laser (TFL) (e.g., early solid-state or hybrid thulium platforms, combined Ho:Thu systems, Nd:YAG, combined Ho and Nd:YAG, 1470 nm diode system). The Thu:YAG category comprises both continuous-wave and pulsed thulium:YAG laser systems.

Earlier studies, predominantly published between 2000 and 2010, mainly used Ho:YAG and Nd:YAG laser technologies or their combinations. Progressive adoption of Thu:YAG platforms began around 2010, while TFL systems were introduced into clinical eKSS practice after 2017 (Figure 3).

Ho:YAG alone was used in four studies [16,21,22,23]. Thu:YAG alone was reported in six studies, including continuous-wave and pulsed systems [15,20,23,25,28,29]. TFL was employed in three studies [26,27,38]. Combination laser platforms included Ho:YAG together with Thu:YAG in four studies [18,30,34,38], and Nd:YAG together with Ho:YAG in four studies [14,31,32,33]. A 1470 nm diode laser system was evaluated in one study [17].

Fiber diameter selection was related to laser technology. Nd:YAG systems mainly employed fibers between 200 and 600 μm, whereas Ho:YAG and Thu:YAG platforms primarily used fibers between 200 and 365 μm. Reporting of power settings was heterogeneous across studies. Among the included series, thulium-derived laser sources generally operated at lower power levels, with Tm:YAG generally used at 5–20 W (occasionally up to 30 W), and TFL commonly between 10 and 50 W. Ho:YAG systems most frequently employed settings corresponding to approximately 0.4–1 J at 10–15 H, resulting in a mean delivered power of about 30–35 W. Nd:YAG operated between 15 and 45 W, while no data are provided for diode laser (Table 2).

### 3.4. Oncological Outcomes

#### 3.4.1. UTUC Recurrence

Ho:YAG-based series demonstrated a weighted recurrence proportion of 51.5% (95% CI: 45–57.2%) at a median pooled follow-up of 34 months, with wide inter-study variability. Thu:YAG-based series included overall showed a lower weighted recurrence proportion of 27.1% (95% CI: 20.9–34.1%) at a median pooled follow-up of 23 months.

**Table 2:** Laser details and outcome of study included in the systematic review.

Study	Laser Type and Pulse Shape	Fiber Diameter	Power Settings	UTUC Recurrence	Bladder Recurrence	Progression	Conversion to RNU	2-yrs RFS	5-yrs RFS	5 yrs-DSS	5-yrs-OS	Any Complication	Major Complications (CD ≥ 3)	Ureteral Stricture	Bleeding (Intervention)	Infection/Fever/Sepsis	Readmission
**Defidio (2019)** **[18]**	Tm-Ho:YAG duo laser (Revolix duo, LisaLaser); CW + PW	270	10–15 W	31/101 (30.7%)	NR/NR	16/101 (15.8%)	9/101 (8.9%)	NR	NR	NR	NR	10/101 (9.9%)	0/101 (0.0%)	NR/NR	0/101 (0.0%)	NR/NR	NR/NR
**Niţă** **(2012)** **[37]**	Nd:YAG; PW	600 or 200	20–45 W	31/62 (50.0%)	20/65 (30.8%)	NR/NR	18/65 (27.7%)	NR	52.3%	NR	NR	NR/65	NR/65	NR/65	NR/65	NR/65	NR/65
**Defidio (2011)** **[38]**	Tm-Ho:YAG duo laser (Revolix duo, LisaLaser); CW + PW	200–365 (200 μm for flexible; 365 μm for semirigid when possible) 200–365 (200 μm for flexible; 365 μm for semirigid when possible)	10–15 W	Overall urothelial recurrence rate 37.3% at median follow-up 26.4 months.	NR	NR	NR	NR	NR	NR	NR	NR	NR	NR	NR	NR	NR
**Tada (2010)** **[14]**	Ho:YAG and/or Nd:YAG (combined depending on size); PW	200	NR	5/15 (33.3%)	6/15 (40.0%)	1/15 (6.7%)	3/15 (20.0%)	0.6	NR	NR	NR	1/15 (6.7%)	1/15 (6.7%)	NR	NR	NR	NR
**Li (2025)** **[17]**	1470 nm diode laser system (Qizhi Corp, China); CW	NR	Cut 120 W; Coagulation 80 W	2/15 (13.3%)	0/15 (0.0%)	1/15 (6.7%)	2/15 (13.3%)	NR	NR	NR	NR	3/15 (20.0%)	0/15 (0.0%)	NR	NR	NR	NR
**Geavlete (2024)** **[22]**	NBI-assisted fURS Ho:YAG, PW	275	35 W	9/61 (14.8%)	NR/NR	NR/61	4/61 (6.6%)	NR	NR	93.4%	NR	NR	NR	NR	NR	NR	NR
	White light Ho:YAG, PW	275	35 W	16/61 (26.2%)	NR/NR	NR/61	6/61 (9.8%)	NR	NR	86.9%	NR	NR	NR	NR	NR	NR	NR
**Hsieh (2020)** **[25]**	Tm:YAG (Quanta Cyber TM 200 W thulium laser), CW	200 (renal pelvis), 365 (ureter)	10–15 W	15/34 (44.1%)	7/34 (20.6%)	2/34 (5.9%)	1/34 (2.9%)	ureter 49%, renal pelvis 60%	NR	2 y: ureter 87%, renal pelvis 100%	NR	5/34 (14.7%)	NR	5/34 (14.7%)	NR	NR	NR
**Wen (2018)** **[28]**	Tm:YAG (Vela XL, StarMedTec), CW/PW	200–600	30–50 W	7/32 (21.9%)	NR/32	0/32 (0.0%)	3/32 (9.4%)	NR	NR	NR	NR	NR	NR	4/32 (12.5%)	4/32 (12.5%)	NR	NR
**Territo (2025)** **[27]**	TFL (Soltive Premium/Pro, Olympus), PW	200	NR	6/33 (21.1%)	NR/NR	1/33 (3.0%)	5/33 (15.2%)	NR	NR	NR	NR	8/33 (24.2%)	1/33 (3.0%)	NR	NR	2/33 (6.1%)	4/33 (12.1%)
**Villa (2018)** **[19]**	Ho:YAG, PW	NR	NR	70/92 (76.1%)	NR/92	31/92 (33.7%)	21/92 (22.8%)	PFS 24 mo: 77%	NR	NR	NR	NR	NR	NR	NR	NR	NR
**Proietti (2022)** **[26]**	TFL (FiberDust, Quanta System), PW	200	10 W (1 J × 10 Hz)	7/28 (25.9%)	NR	NR	NR	NR	NR	NR	NR	4/28 (14.2%)	0/28 (0.0%)	0/28 (0.0%)	NR	NR	NR
**Bozzini (2021)** **[15]**	Tm:YAG (Cyber TM, Quanta), CW	272	15–30 W renal pelvis; 15 W ureter	9/47 (19.1%)	7/47 (14.9%)	NR	1/47 (2.1%)	NR	NR	NR	NR	21/47 (44.7%)	0/47 (0.0%)	0/78 (0.0%)	0/78 (0.0%)	9/78 (11.5%)	NR/78
**Matsuoka (2003)** **[32]**	Ho:YAG/Nd:YAG, PW	365 (ureter/pelvis/upper calyx); 200 (mid/lower calyx)	Nd:YAG 15–40 W × 2–3 s (2/33 cases)	10/27 (37.0%)	5/27 (18.5%)	3/27 (11.1%)	NR	NR	NR	NR	NR	1/33 (3.0%)	NR	1/33 (3.0%)	NR	NR	NR
**Ye (2025)** **[20]**	Tm:YAG (SRM-T120F, Youlu), CW	NR	5–20 W	12/33 (36.4%)	4/33 (12.1%)	0/33 (0.0%)	2/33 (6.1%)	64%	NR	NR	NR	NR	1/33 (3.0%)	4/33 (12.1%)	NR	3/33 (9.1%)	NR
**Cornu (2010)** **[16]**	Ho:YAG, PW	200	NR	21/35 (60.0%)	14/35 (40.0%)	0/35 (0.0%)	4/35 (11.4%)	NR	NR	100%	NR	3/35 (8.6%)	NR	NR	NR	2/35 (5.7%)	NR
**Hubosky (2013)** **[31]**	Ho:YAG/Nd:YAG, PW	NR	NR	3/15 (2–5.0%)	7/13 (53.8%)	1/13 (7.7%)	1/15 (6.7%)	NR	NR	NR	NR	4/15 (26.7%)	NR	4/15 (26.7%)	NR	NR	NR
**Kalaitzis (2013)** **[21]**	Ho:YAG, PW	365	NR	24/25 (96.0%)	7/25 (28.0%)	2/25 (8.0%)	1/25 (4.0%)	NR	NR	NR	NR	NR	NR	3/25 (12.0%)	11/25 (44.0%)	1/25 (4.0%)	NR/25
**Musi (2018)** **[23]**	Tm:YAG, CW	272 µm (flexible); 365 µm (semirigid ureteral lesions)	10–20 W (sometimes 5 W for coagulation)	8/42 (19.0%)	NR	NR	4/42 (9.5%)	NR	NR	NR	NR	37/42 (88.1%)	1/42 (2.4%)	0/42 (0.0%)	1/42 (2.4%)	NR	NR
**Proietti (2021)** **[34]**	Ho:YAG and Tm:YAG, PW	NR	NR	18/29 (61.1%)	9/29 (31.0%)	1/29 (3.4%)	1/29 (3.4%)	31.7% (±9.4%)	NR	NR	96.4 ± 3.5%	20/137 (14.6%, per procedure)	4/137 (2.9%, per procedure)	0/137 (0%)	NR	NR	NR
**Sanguedolce (2021)** **[30]**	Ho:YAG and Tm:YAG, PW	NR	Ho:YAG 0.8–1.2 J; 8–12 Hz|Tm:YAG 10–15 W	13/47 (28.3%)	11/47 (23.4%)	9/47 (19.1%)	8/47 (17.0%)	NR	NR	NR	NR	5/47 (10.6%)	0/47 (0.0%)	1/47 (2.1%)	1/47 (2.1%)	3/47 (6.4%)	NR
**Shvero (2021)** **[33]**	Mixed (Ho:YAG and Nd:YAG), PW	NR	NR	46/62 (74.2%, per procedure)	27/59 (45.8%)	4/59 (6.8%)	3/59 (5.1%)	NR	NR	NR	NR	16/59 (27.1%)	5/59 (8.5%)	NR/59	2/59 (3.4%)	5/59 (8.5%)	16/59 (27.1%)
**Yoshida (2021)** **[24]**	first arm Tm:YAG and Ho:YAG + PDD (5-ALA); PW + CW. Second arm Ho:YAG; PW	272 272	Tm:YAG 5 W/15 W; Ho:YAG 0.4 J/15 Hz long pulse + 1 J/10 Hz short pulse	NR NR	NR NR	NR NR	0/10 (0%) 8/16 (41.3%)	57.1% NR	NR NR	NR NR	NR NR	10/10 (100%) 16/16 (100%)	0/10 (0.0%) 1/16 (1%)	0/10 (0%) 1/16 (6%)	NR NR	1/10 (10%) 3/16 (18%)	NR NR
**Figaroa (2024)** **[35]**	Ho:YAG, PW	NR	1 J/10 Hz	NR	NR	NR	23/71 (32.4%)	22%	NR	86%	82%	NR	NR	NR	NR	NR	NR
**Proietti (2025)** **[29]**	p-tm:YAG (Dornier p-tm:YAG) PW	200	NR	NR	NR	1/20 (5.0%)	1/20 (5.0%)	NR	NR	NR	NR	10/20 (13.3%)	0/20 (0.0%)	0/20 (0.0%)	NR	NR	NR
**Bislev (2024)** **[36]**	TFL (Soltive, Olympus), PW	150	NR	83/118 (70.3%)	52/118 (44.1%)	NR	20/118 (16.9%)	28.8%	NR	94.1%	59.1%	NR	NR	NR	NR	NR	NR

Abbreviation: PW, pulsed wave; CW, continuous wave; URS, ureteroscopy; fURS, flexible ureteroscopy; UTUC, upper tract urothelial carcinoma; RNU, radical nephroureterectomy; RFS, recurrence-free survival; PFS, progression-free survival; DSS, disease-specific survival; OS, overall survival; NBI, narrow band imaging; PDD, photodynamic diagnosis; 5-ALA, 5-aminolevulinic acid; Ho:YAG, holmium:yttrium–aluminum–garnet laser; Tm:YAG, thulium:yttrium–aluminum–garnet laser; p-Tm:YAG, pulsed thulium:YAG laser; TFL, thulium fiber laser; Nd:YAG, neodymium:yttrium–aluminum–garnet laser; CD, Clavien–Dindo classification; NR, not reported.

**Figure 4:**
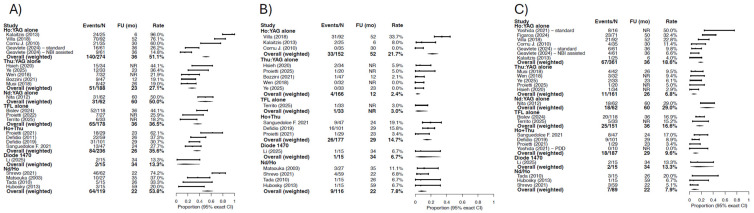
Descriptive forest plots of oncological outcomes stratified by laser technology: (**A**) recurrence, (**B**) progression, and (**C**) radical nephroureterectomy (RNU). For each study, proportions are reported as events over the corresponding denominator, with exact 95% confidence intervals (Clopper–Pearson). Within each laser category, an overall weighted estimate is provided, calculated by aggregating event counts and denominators across studies. Follow-up duration (months) is reported when available; studies reporting follow-up as not reported (NR) were retained and treated as missing for follow-up summaries. When oncological outcomes were reported per procedure rather than per patient, the procedure-level data were used as reported by the original study. No formal meta-analytic pooling or heterogeneity assessment was performed.

Among TFL-based studies, the weighted recurrence proportion was approximately 36.5% (29.4–44.0%) at median follow-up of 36 months. Series employing combined Ho:YAG and Thu:YAG platforms demonstrated a weighted recurrence of 35.6% (29.5–42.1%). Studies using combined Nd:YAG and Ho:YAG systems showed a weighted recurrence proportion of 53.8% (95% CI: 44.4–63.0). Considerable dispersion in recurrence rates was observed across studies and laser categories (Figure 4).

#### 3.4.2. Progression Rate and Conversion to RNU

Progression events were uncommon across most laser categories. Ho:YAG-based series demonstrated the highest weighted progression proportion among subgroups, assessed at 21.7% (95% CI: 15.4–29.1%). Thu:YAG-based series showed the lowest weighted progression, assessed at 2.4% (95%CI: 0.7–6.1%) at a median follow-up of 12 months. TFL-based studies reported a similar progression rate, with a weighted proportion of 3.0% (0.1–15.8%). Studies using combined Nd:YAG and Ho:YAG systems demonstrated a weighted progression proportion of 7.8% (95%CI: 3.6–14.2%) (Figure 4) at a median follow-up of 22 months. Combined Ho:YAG and Thu:YAG platforms were associated with a weighted progression proportion of 14.7% (95%CI: 9.8–20.8%).

Overall, progression rates were lowest in Thu:YAG and TFL series.

Rate of conversion to RNU varied across laser categories. Ho:YAG alone was associated with a weighted RNU rate of 18.6% (95%CI: 14.7–23.0) at a median pooled follow-up of 36 months. TFL-based studies showed a weighted RNU proportion of 16.6% (95% CI: 11.0–23.5%). Combined Ho:YAG and Thu:YAG platforms demonstrated a weighted RNU rate of 9.6%, while the lower weighted RNU proportion was observed in Thu:YAG-based series (6.8% at 26 months).

Thu:YAG and combined Ho:YAG and Thu:YAG categories demonstrated numerically lower progression and RNU rates compared with Ho:YAG-based series; however, median follow-up across these studies was tendentially shorter than in the TFL and Ho:YAG series.

### 3.5. Complications Outcomes

#### 3.5.1. Overall eKSS-Related Events

Ho:YAG-based series demonstrated a weighted any-complication rate of approximately 39.5% (95% CI: 28.4–51.4%). Thu:YAG-based studies showed a weighted rate of approximately 41.3% (95%CI: 34.6–48.4%), whereas TFL-based series demonstrated a weighted rate of approximately 19.7% (95%CI: 10.6–31.8%).

**Figure 5:**
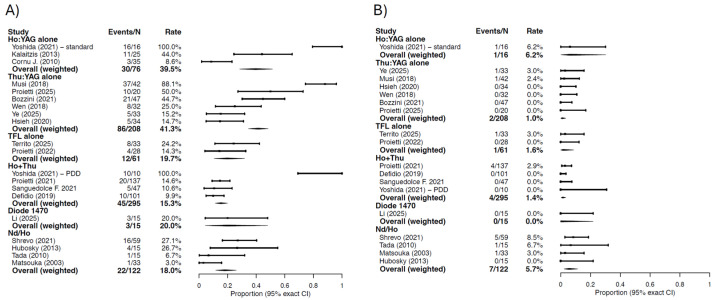
Descriptive forest plots of complications according to Clavien–Dindo: (**A**) any complication, (**B**) major complications (CD ≥ 3). For each study, proportions are reported as events over the corresponding denominator, with exact 95% confidence intervals (Clopper–Pearson). Within each laser category, an overall weighted estimate is provided, calculated by aggregating event counts and denominators across studies. Events are expressed as ratio between number of occurrences per study and total treatments. When oncological outcomes were reported per procedure rather than per patient, the procedure-level data were used as reported by the original study. No formal meta-analytic pooling or heterogeneity assessment was performed.

Combined Ho:YAG and Thu:YAG platforms were associated with a weighted any-complication rate of approximately 15.3%. A single diode laser study reported an overall complication rate of 20.0%. Combined Nd:YAG and Ho:YAG systems demonstrated a weighted any-complication proportion of approximately 18.0% (Figure 5).

Minor complications predominantly consisted of transient postoperative pain requiring analgesic therapy and, in cases of suspected urinary tract infection or fever, administration of broad-spectrum antibiotics. In some cases, with perioperative bleeding or persistent hematuria despite ureteral stenting, blood transfusion was required to maintain hemodynamic stability.

#### 3.5.2. Major Events

Major complications (Clavien–Dindo grade ≥ III) were rare across all laser categories. Weighted major complication rates were approximately 6.2% (0.2–30.2%) for Ho:YAG alone, 1.0% (0.1–3.4%) for Thu:YAG alone, 1.6% (95%CI: 0–8.8%) for TFL alone, 1.4% (95%CI: 0.4–3.4%) for combined Ho:YAG and Thu:YAG platforms, and 5.7% (95%CI: 2.3–11.5%) for combined Nd:YAG and Ho:YAG systems. No major complications were reported in the single diode laser study (Figure 5).

Major adverse events most commonly consisted of early upper urinary tract drainage with placement of a double-J ureteral stent in the setting of hydronephrosis associated with acute kidney injury or sepsis. In sporadic cases, patients required admission to an intensive or high-dependency care unit for management of acute renal failure requiring hemodialysis or for treatment of septic shock.

## 4. Discussion

In this systematic review, we provide a comprehensive descriptive synthesis of laser technologies used for eKSS in patients with UTUC, stratifying oncological and safety outcomes according to the laser energy source. Given the pronounced clinical and methodological heterogeneity across available studies, including differences in patient selection, tumor risk profile, endoscopic technique, outcome definitions, and follow-up duration, we deliberately adopted a descriptive, sample-size-weighted approach and refrained from formal meta-analytic pooling [13,39].

### 4.1. Summary of Findings

Three consistent messages emerge from our analysis.

First, local upper tract recurrence remains frequent after eKSS, irrespective of the laser technology employed. Except for the diode laser series, weighted recurrence proportions ranged from approximately 30% to over 50% across laser categories, with wide inter-study variability. This observation confirms that recurrence represents a fundamental limitation of conservative UTUC management and is likely driven by tumor biology, multifocality, and field cancerization rather than by the intrinsic performance of a specific laser platform. In particular, Thu:YAG-based series demonstrated a lower weighted recurrence proportion compared with Ho:YAG-based series (27.1% vs. 51.1%), while TFL-based series showed an intermediate recurrence proportion (36.5%).

Second, disease progression and conversion to RNU were relatively uncommon in the series using thulium-derived technologies (2.4% and 6.8%, respectively). While TFL systems showed a similar weighted pathology progression (3%), pulsed TFL systems had a greater rate of RNU (16.6%) compared to thu:YAG. Similarly, Ho:YAG-based cohorts reported a weighted RNU proportion of 18.6%, and the highest weighted progression proportion (21.7%) among all the laser energies.

These findings suggest that, although recurrence is common, biological progression leading to loss of kidney-sparing feasibility occurs in a minority of patients when treated with thulium-based systems, supporting the oncological acceptability of eKSS in selected settings [40]. Pulsed TFL-based system showed intermediate results in terms of oncological outcomes between Thu:YAG and Ho:YAG systems.

Third, major complications were rare across all laser technologies, with broadly comparable safety profiles. Minor complications were more frequently reported but highly heterogeneous in definition and reporting, limiting meaningful comparisons between laser platforms. Although Thu:YAG systems showed a relatively high weighted rate of any complication (41.3%), the corresponding weighted rate of major complications was only 1%.

A tendency toward higher major complication rates was observed in Nd:YAG- and Ho:YAG-based series. These results are consistent with the historical literature. In the series by Blute et al. [41] patients were treated mainly with Nd:YAG laser at power settings between 30 and 35 W and major complication rates of approximately 7%. Accordingly, Nd:YAG + Ho:YAG series demonstrated the highest weighted proportion rate of major complication with Ho:YAG series (5.7% and 6.2%). This may reflect the limited controllability of Nd:YAG energy, which exhibits deep tissue penetration approaching 10 mm due to hemoglobin as the primary chromophore [37,42].

### 4.2. Historical and Technological Context

The evolution of eKSS for UTUC reflects a progressive refinement of energy sources aimed at balancing effective tumor ablation with preservation of the urothelial wall. Early conservative experiences relied on percutaneous resection, ureteroscopic fulguration, and Nd:YAG laser ablation [43,44,45].

Despite the less favorable trends observed for Nd:YAG-based series in our systematic review, some earlier cohorts reported acceptable oncological outcomes with this energy source when coupled with strict surveillance protocols.

For instance, Gaboardi et al. treated 18 patients with histologically confirmed UTUC using retrograde ureteropyeloscopic Nd:YAG laser irradiation (25–30 W), reporting that ten patients remained tumor-free after 6–30 months of follow-up, while eight experienced recurrence managed with repeat laser ablation and intracavitary chemotherapy [46]. Only one patient required subsequent RNU, and no cases of disseminated disease were observed.

The subsequent introduction of Ho:YAG laser represented a pivotal technical advance [47]. Its shallow penetration depth and water-based absorption improved safety and precision, facilitating widespread adoption in eKSS. Large Ho:YAG-based series have consistently reported satisfactory cancer-specific survival, particularly in low-grade tumors, while documenting high rates of both local recurrence and progression. Although recurrence and progression appear to follow similar trends within Ho:YAG-only cohorts, comparison across different laser platforms suggests that these two endpoints may represent biologically distinct phenomena [18,19,32].

Thulium-based platforms were introduced with the goal of further improving ablation precision and hemostasis. Continuous-wave and p-Thu:YAG systems provide continuous or quasi-continuous energy delivery with high water absorption and limited penetration depth, potentially enhancing visualization and completeness of ablation. In the present review, series employing Thu:YAG and pulsed systems (TFLs) demonstrated lower weighted progression and RNU rates compared with Ho:YAG-based cohorts, suggesting a possible oncological advantage in terms of biological control rather than local recurrence prevention. Thu:YAG-based systems may exhibit a greater ability to achieve effective tissue vaporization compared with Ho:YAG, which, in the context of UTUC treatment, could translate into improved oncological outcomes. Furthermore, patients treated with TFL and Thu:YAG systems showed a lower weighted rate of overall complications, which may reflect the superior cutting precision and coagulation properties of thulium-based energy compared with continuous-wave Ho:YAG systems.

### 4.3. Interpretation and Sources of Bias

Despite these encouraging descriptive trends, caution is warranted in attributing improved outcomes to laser technology alone. Several sources of bias likely influence the observed differences. Most notably, temporal confounding is substantial: Thu:YAG and TFL series are predominantly more recent and therefore benefit from parallel advances in digital flexible ureteroscopy, high-definition optics, irrigation systems, biopsy devices, perioperative management, and structured surveillance protocols [48]. In addition, patient selection and risk stratification have evolved over time, with increasing adherence to guideline-based criteria favoring conservative management in biologically favorable tumors [3,49].

Follow-up duration represents another critical limitation. Median follow-up in thulium-based and TFL series was generally shorter than in Ho:YAG cohorts, potentially underestimating late progression and RNU rates. Given the indolent but persistent natural history of UTUC, particularly in high-grade disease, short follow-up may artificially accentuate apparent differences between laser platforms.

Furthermore, outcome definitions varied widely across studies. Recurrence, progression, and endoscopic failure were inconsistently defined, and outcomes were often reported per procedure rather than per patient. Reporting of laser settings, fiber diameter, and ablation strategy was incomplete and non-standardized, precluding meaningful assessment of dose–response relationships or technical determinants of oncological control.

### 4.4. Role of Tumor Biology and Patient Selection

Across all laser technologies, tumor biology remains the dominant driver of oncological outcomes [50]. Low-grade UTUC treated endoscopically is consistently associated with excellent disease-specific survival despite frequent recurrences, whereas high-grade disease carries a substantially higher risk of progression, metastasis, and loss of kidney-sparing feasibility. In the present review, a considerable proportion of patients harbored high-grade and/or high-risk tumors, often treated under imperative indications, which likely contributed to the observed heterogeneity in progression and RNU rates.

These findings reinforce current guideline recommendations that endoscopic eKSS should primarily be reserved for low-risk UTUC, while conservative treatment of high-risk disease should be limited to selected imperative cases managed in experienced centers and within strict surveillance protocols [3].

### 4.5. Clinical Implications

From a clinical standpoint, current evidence does not support the identification of a single “best” laser platform for UTUC eKSS. While thulium-derived technologies and TFL show favorable descriptive trends, these data remain hypothesis-generating and insufficient to justify a change in standard practice. Laser selection should therefore be individualized based on tumor characteristics, procedural objectives, surgeon expertise, and institutional availability, rather than expectations of superior oncological efficacy related to laser type alone. The major knowledge gap highlighted by this review is the absence of high-quality comparative evidence. Future studies should prioritize prospective multicenter designs or robust registries with standardized eligibility criteria, harmonized outcome definitions, adequate follow-up, and detailed reporting of laser parameters and ablation techniques. Only through such methodologically rigorous efforts will it be possible to determine whether specific laser technologies confer clinically meaningful advantages in oncological control or safety in kidney-sparing management of UTUC.

## 5. Limitations

Some limitations of the present systematic review should be acknowledged. First, the available evidence is almost exclusively based on retrospective, single-center observational studies, with moderate-to-serious risk of bias, particularly related to confounding and patient selection. No randomized controlled trials and only a limited number of prospective cohorts were identified, which precludes causal inference.

Second, substantial heterogeneity exists across studies in terms of patient risk profiles, tumor grade and stage distribution, multifocality, lesion size, endoscopic techniques, laser settings, fiber diameters, and follow-up schedules. Outcome definitions, especially for recurrence, progression, and endoscopic failure, varied considerably, limiting comparability. Many studies reported outcomes per procedure rather than per patient, and intravesical recurrence and survival endpoints were inconsistently available.

Third, several series reported the use of newer Thu:YAG systems in pulsed modality, such as pulsed p-tm:YAG, as well as platforms combining continuous-wave Tm:YAG with Ho:YAG. Given the limited number of studies within each subgroup and the substantial heterogeneity in pulse-modulated technologies, we did not perform separate analyses based on pulse shape. Instead, authors elected to prioritize classification according to solid-state laser technology (energy source) rather than emission mode. This represents a reflection that new technologies might support even more promising outcomes than those shown in this review as more evidence becomes available.

Furthermore, the reporting of laser-related technical parameters was incomplete and non-standardized in most studies, preventing meaningful assessment of dose–response relationships or identification of optimal settings. Similarly, advances in endoscopic instrumentation and imaging over time introduce relevant temporal confounding, particularly when comparing older Nd:YAG or Ho:YAG series with more recent thulium-based cohorts.

Finally, although we used sample-size-weighted descriptive estimates, this approach does not account for between-study variability and should not be interpreted as formal pooled effect measures. The observed differences among laser categories must therefore be considered hypothesis-generating.

## 6. Conclusions

Endoscopic kidney-sparing laser treatment represents a feasible and safe strategy for selected patients with upper tract urothelial carcinoma. Across heterogeneous observational evidence, Thu:YAG and TFL platforms seem to demonstrate encouraging descriptive trends toward lower recurrence, progression rates and major complications. However, due to the heterogeneity of reports and temporal trends toward the implementation of these technologies, in more recent reports superiority cannot be inferred.

Endoscopic eKSS using laser energy should be regarded as the cornerstone endourological approach when UTUC patients are appropriately selected. Particular interest should be placed in thulium-derived platforms, based on their favorable descriptive oncological outcomes and low complications rate. However, in the absence of high-level comparative evidence, no standard laser platform can be recommended based on existing data. Prospective, comparative, and methodologically robust studies are needed to define whether specific laser technologies confer clinically meaningful advantages in oncological control or safety in UTUC eKSS.

## Supplementary Materials

The following supporting information can be downloaded at https://www.mdpi.com/article/10.3390/cancers18050821/s1; Supplementary File S1: Search strategy string; Supplementary File S2: PRISMA 2020 Checklist; Supplementary Figure S1: Risk of bias assessment.
