# Supplementary material for: Laser Energy Application in Endoscopic Kidney-Sparing Surgery for Upper Tract Urothelial Carcinoma: A Systematic Review of Oncological Outcomes and Surgical Complications
Source: Cancers (Basel). 2026 Mar 3;18(5):821. doi: 10.3390/cancers18050821 (PMC12984495; doi:10.3390/cancers18050821)
Supplement: Supplementary file 1 [file cancers-18-00821-s001.zip › sFile_1.pdf]

Search strategy string

```
(
 (
  "Ureteral Neoplasms"[Mesh]
  OR "Renal Pelvis Neoplasms"[Mesh]
  OR "upper tract urothelial carcinoma"[Title/Abstract]
  OR UTUC[Title/Abstract]
  OR "upper urinary tract carcinoma"[Title/Abstract]
  OR "upper urinary tract urothelial carcinoma"[Title/Abstract]
  OR "ureter neoplasm"[Title/Abstract]
  OR "ureter neoplasms"[Title/Abstract]
  OR "ureteral tumor"[Title/Abstract]
  OR "ureteral carcinoma"[Title/Abstract]
 )
 AND
 (
  "Kidney Sparing Surgery"[Title/Abstract]
  OR "kidney-sparing"[Title/Abstract]
  OR "nephron-sparing"[Title/Abstract]
  OR endoscopic[Title/Abstract]
  OR ureteroscopic[Title/Abstract]
  OR ureteroscopy[Mesh]
  OR "endoscopic management"[Title/Abstract]
 )
 AND
 (
  laser[Title/Abstract]
  OR "laser ablation"[Title/Abstract]
  OR "laser fulguration"[Title/Abstract]
  OR holmium[Title/Abstract]
  OR "Ho:YAG"[Title/Abstract]
  OR "holmium:YAG"[Title/Abstract]
  OR thulium[Title/Abstract]
  OR "thulium:YAG"[Title/Abstract]
  OR "thulium fiber laser"[Title/Abstract]
  OR TFL[Title/Abstract]
 )
)
AND ("2000/01/01"[Date - Publication] : "3000"[Date - Publication])
```
