# Supplementary figures and images for: Laser Energy Application in Endoscopic Kidney-Sparing Surgery for Upper Tract Urothelial Carcinoma: A Systematic Review of Oncological Outcomes and Surgical Complications
Source: Cancers (Basel). 2026 Mar 3;18(5):821. doi: 10.3390/cancers18050821 (PMC12984495; doi:10.3390/cancers18050821)

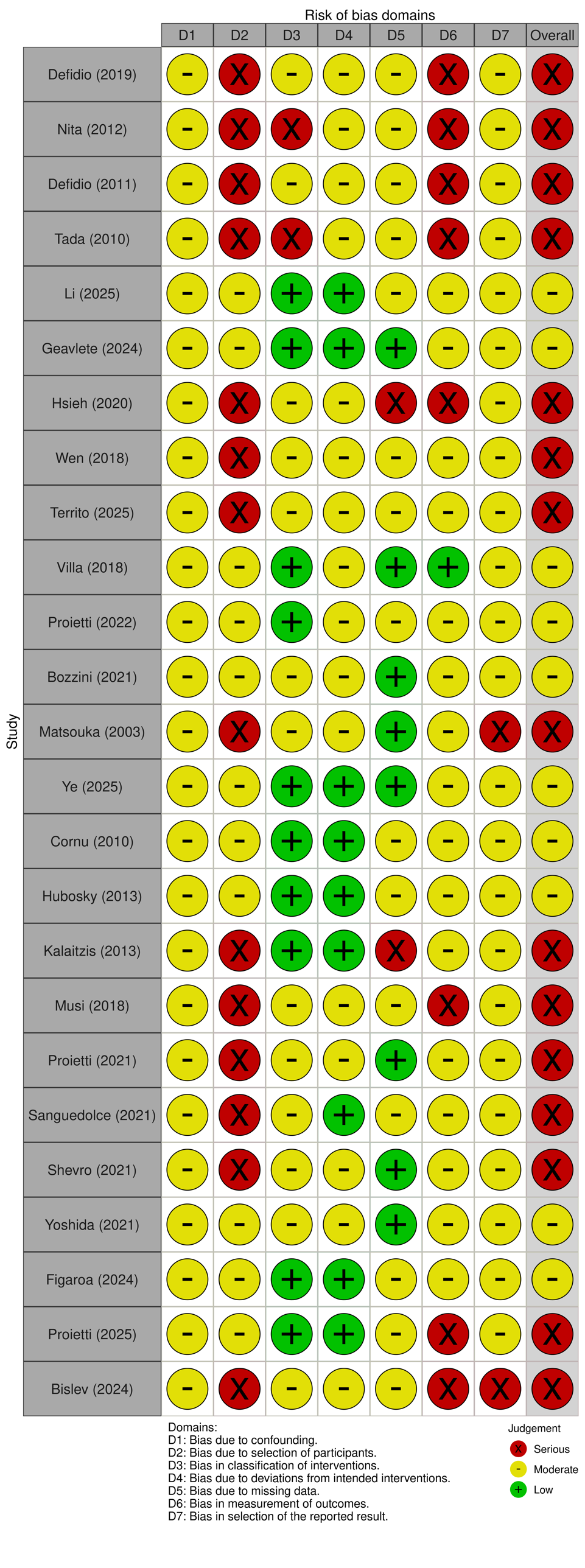

Supplement: Supplementary file 1 [file cancers-18-00821-s001.zip › sFigure_1.png]
